# Can family planning outreach bridge the urban-rural divide in Zambia?

**DOI:** 10.1186/1472-6963-7-143

**Published:** 2007-09-05

**Authors:** Justin S White, Ilene S Speizer

**Affiliations:** 1 MEASURE Evaluation Project, Carolina Population Center, The University of North Carolina at Chapel Hill, Chapel Hill, NC; 2 Department of Maternal and Child Health, The University of North Carolina at Chapel Hill, School of Public Health, Chapel Hill, NC

## Abstract

**Background:**

Zambia experienced declining aggregate fertility and increasing aggregate contraceptive use from 1990 to 2000. Yet, in rural Zambia, progress in family planning has lagged far behind the advances made in Zambia's urban areas. The contraceptive prevalence rate in Lusaka and other urban areas outstripped the rate in rural Zambia by nearly 25 percentage points (41.2 percent versus 16.6 percent) in 2001. The total fertility rate varied between urban and rural areas by 2.5 children (4.3 versus 6.9 children). This paper considers the urban-rural differentials in Zambia and assesses family planning outreach as a tool to narrow this divide.

**Methods:**

This study uses the Zambia Demographic and Health Survey (DHS) data, collected between 2001 and 2002. Logistic regression techniques were employed to examine factors associated with contraceptive use. The first analysis tested modern contraceptive use versus traditional method use and no use. In addition, separate models were run for samples stratified by type of residence (rural or urban) to determine if different factors were associated with use by residence. A simulation determined the effect of all women receiving at least one household visit from a health worker if all other variables were held constant.

**Results:**

Differences in modern contraceptive use between urban and rural areas persist (OR: 1.56, 95 percent CI: 1.24–1.96) even after adjusting for a number of demographic, socioeconomic, cognitive, and attitudinal factors. Household visits by a community health worker significantly increased the likelihood of modern contraceptive use among rural women (OR: 1.83; 95 percent CI: 1.29–2.58). If all rural women received at least one outreach visit per year, the prevalence rate for modern contraceptive methods would be expected to increase for this group by 5.9 percentage points, a marked increase but less than one-quarter of the total urban-rural differential.

**Conclusion:**

Outreach in the form of health worker visits can improve access to family planning services, but it does not eliminate barriers to access or address continued high-fertility desires in Zambia. Until policymakers consider strategies that address both family planning demand creation and supply of services, progress in Zambia and the rest of sub-Saharan Africa will continue to lag behind the rest of the world.

## Background

Family planning has been proven to save and enhance the lives of women, children, and families. It reduces the number of unintended, unwanted, and mistimed pregnancies. Women who control their fertility have fewer unsafe abortions, thereby saving mothers' lives. Family planning allows women to space births, and longer birth intervals reduce maternal and infant mortality rates [1,2]. Family planning and birth spacing also reduce unwanted pregnancy among HIV-positive women, which limits the number of infants born with HIV [3]. Yet, family planning as a health and development strategy has not been promoted consistently everywhere. Low rates of contraceptive use and high fertility rates persist in most countries of sub-Saharan Africa [4,5].

In addition to large differences in use of family planning across sub-regions of Africa, wide variation exists within countries. In particular, sub-Saharan Africans living in rural areas tend to use fewer contraceptives and have more children than their urban counterparts [1,6]. A recent study concluded that the rural-urban differential in fertility is an inequity, reflecting an inability of poor, rural women to achieve their desired fertility, rather than an inequality in which rural women simply want large families [7]. Community-based distribution (CBD) of family planning is one of the most common strategies used to reach populations with limited access to services, especially in rural areas. The approach uses non-professional local workers, sometimes volunteers, who live in or visit communities to provide services that a woman otherwise would have to travel to obtain [8]. CBD also involves sharing knowledge about the importance of family planning services and the proper use of family planning; this information may alter couples' fertility intentions, a factor that influences the demand for family planning services [7]. Overall, the CBD strategy assumes that lack of convenient access to contraceptives represents a substantial barrier to meeting the needs of family planning users.

In the 1970s, widespread use of CBD occurred in Asia and Latin America, but the first programs did not reach Africa until the early 1980s [9]. The popularity of CBD programs in Africa grew steadily throughout the next two decades–from seven countries operating programs in 1984 to 20 countries between 1994 and 1998 [10]. The evidence evaluating the success of CBD efforts has been mixed. Two of the best-documented initiatives employing household visits from community health workers were based in rural Bangladesh and in rural Ghana. Sustained outreach efforts in Bangladesh led to continued uptake of family planning services even after a decade of health worker visits [11]; however, the country's subsequent shift from household distribution of family planning services to clinic-based service delivery proved at least as effective, with contraceptive prevalence increasing slightly after the shift [12]. In the Navrongo experiment, from 1993 to 1999, household visits conducted by trained nurses in communities of northern Ghana significantly increased the likelihood that married women prefer both to limit and to space births compared to either wanting more children soon or being unsure about fertility preferences [13]. However, the researchers found that increased contraceptive use was not a primary determinant of the fertility decline.

A systematic review of CBD in Africa concluded from several quasi-experimental and descriptive studies that community distributors could increase contraceptive use, although the level of effects are often unknown or less than reported for similar projects conducted in Asia [10]. Prata and colleagues (2005) reviewed the evidence on CBD programs and concluded that CBD serves an important role in meeting the needs of rural communities [14]. Questions regarding the utility of CBD programs also have been raised, including the cost-effectiveness of delivering goods to remote areas, the sustainability of the community-based model, the inability to scale small pilot projects to regional or national levels, and the long-term effect of CBD on national fertility due to low coverage [10,11,15,16]. Some concerns such as the scalability and sustainability of CBD have been addressed to an extent [11,17].

This study extends our understanding of the potential role of community-based distribution for stimulating family planning use in rural areas of sub-Saharan Africa. Zambia was selected for study because of its differential access to health services between rural and urban areas and its prominence as a 'success story' in a recent case study on family planning use in Africa [18-20]. Multiple studies have underscored the health service gap between rural and urban Zambia. For example, in 2005, a group of reproductive health experts were asked to rate Zambian women's access to a range of reproductive health services, such as postpartum family planning and antenatal care [18]. On a scale from 0–100, with 0 indicating a low score, the mean score on access to reproductive health services was 66 for urban areas and 30 for rural areas. Another study found that, among urban health centers, 88 percent offer voluntary counselling and treatment for HIV/AIDS and 47 percent offer prevention of mother-to-child transmission of HIV/AIDS, whereas the respective proportions are 25 percent and 12 percent among rural health centers [19].

Zambia's success in family planning was documented most recently through a country-specific case study that revealed an impressive aggregate drop in the total fertility rate (TFR) and an aggregate rise in the modern contraceptive prevalence rate (CPR) between the early 1990s and the early 2000s [20]. Modest, sub-national efforts to use community health workers for the delivery of family planning services were believed to be an effective, albeit underused, tool for getting services to rural populations [20]. For example, the Planned Parenthood Association of Zambia, a major implementing agency of CBD programs, integrated community-based family planning and reproductive health with nutrition, education, and income-generating activities in 470 villages of the Copperbelt and Luapula provinces [21]. The CPR in project areas was 46.1 percent, 20 percentage points above the national average at the time of measurement (in 1996) [22]. The full scope of similar programs is not well documented and their impact on national trends for contraceptive use is unknown.

Using quantitative data from Zambia, this study focuses on answering the following three research questions relating to Zambia's success at stimulating modern contraceptive use: 1) Controlling for individual-level socio-economic factors associated with modern family planning use, is location of residence (rural vs. urban) associated with use? 2) Among women in rural areas, are outreach workers associated with modern contraceptive use? And 3) what would be the simulated effect on modern contraceptive use if all rural women had access to family planning outreach?

## Methods

This study uses the Zambia Demographic and Health Survey (DHS) data, collected between 2001 and 2002 [22]. The survey includes a nationally representative sample of 7,658 women aged 15–49. The study used a multistage sampling design that first selected a random sample of enumeration areas and then selected a random sample of households systematically from a household listing of all households in the enumeration area. All eligible women in the sampled households were approached and asked to participate in the interview. Women who consented to be interviewed were asked the survey questions by trained interviewers. Interviewed women answered detailed questions on pregnancy history, family planning, and fertility preferences. Information from the questionnaire provides data on the demographic, socioeconomic, and cultural characteristics of users and non-users of contraception.

The analysis focuses on current fertility intentions and contraceptive use. Thus, the study sample was reduced to include only the 4,927 women (unweighted sample size; weighted value is 4,906) who were exposed to the risk of pregnancy. In particular, women were excluded who were currently pregnant, infecund, or who had not had sex in the last year. Currently pregnant women were excluded because they were not asked about their current contraceptive use. The study sample represents 64 percent of the women in the full sample surveyed in 2001 to 2002.

Background characteristics were selected for inclusion in the analysis based on their significance in previous studies of contraceptive behavior or on their hypothesized association with contraceptive choice. Demographic factors included type of residence (urban vs. rural), age (15–24, 25–34, 35–39, 40–44, 45–49), number of living children (none, 1, 2, 3, 4+), and marital status (never married, currently married or in union, formerly married). Socioeconomic factors included highest educational attainment (no education, primary education, secondary education and higher), employment status (not working vs. currently working), and quintile of socioeconomic status (SES) (very low, low, medium, high, very high). SES was estimated by expressing the possession of selected household assets as one summary variable developed based on the results of principal components factor analysis applied to the individual-level data [23]. Additional factors included in multivariate models were desire for more children (wants more soon, wants more later [in 2+ years], wants more/unsure of timing, wants no more), a woman's attitude toward family planning (approves, disapproves, doesn't know), exposure to family planning on the radio in the last few months (no/yes), and currently amenorrheic (no/yes). Finally, the key independent variable of interest that captures exposure to community-based health workers was whether anyone in the household was visited by a community health worker in the last year (no/yes).

Logistic regression techniques were employed to examine factors associated with contraceptive use. The first analysis tested modern contraceptive use versus traditional method use or no use. Modern contraceptive methods included the contraceptive pill, three-month injectables, female or male condoms, sterilization, diaphragm, and spermicidal foam. Traditional methods included withdrawal, periodic abstinence, the rhythm method, and lactational amenorrhea (which is commonly treated as a modern method but was confused with regular breastfeeding in the survey) [22]. In addition, separate models were run for samples stratified by type of residence (rural or urban) to determine if different factors were associated with use by residence. Point estimates are reported as the odds ratio of use versus non-use (e.g., modern use vs. traditional use or non-use), along with the corresponding 95 percent confidence intervals.

Simulation models were created to predict the change in contraceptive use if all women received a visit from a community health worker, keeping all other variables constant. These were tested in aggregate models and models stratified by type of residence. Outreach workers typically operate in rural areas, which were predicted to have a higher prevalence of visits. Thus, it is hypothesized that if all women received a visit in rural areas there would be a greater increase in contraceptive use than if all women received a visit in urban areas. Simulation models were also created for other factors associated with use, namely the desire to delay future births, the desire to limit future births, and exposure to family planning messages on the radio.

All analyses were adjusted for the multi-stage sampling design and were weighted. All analyses were performed using Stata version 9.2.

## Results

### Descriptive summary

Zambia is a land-locked country in southern Africa, bordered by eight other countries. According to the 2000 census, of the 10.3 million people living in Zambia, 36 percent of the population resides in urban areas, heavily concentrated in the provinces of Copperbelt and Lusaka [24]. Between 1992 and 2001–02, the total fertility rate (TFR) in Zambia fell more than half a child per woman (from 6.5 to 5.9) and the contraceptive prevalence rate (CPR) more than doubled (Table 1). However, the aggregate picture masks important underlying disparities between rural and urban areas. Urban areas enjoyed substantial decreases in TFR (from 5.8 to 4.3) and increases in CPR (modern: 15.3 percent to 41.2 percent). In rural areas contraceptive use increased and fertility declined at a faster rate, but of a much smaller magnitude than in urban areas (TFR: 7.1 to 6.9; CPR: 3.2 percent to 16.6 percent). Moreover, the gains in CPR for rural areas were due in part to take-up of less effective, traditional methods of contraception [25].

**Table 1:** Total fertility rate (TFR) and contraceptive prevalence rate (CPR) in Zambia, by year and type of residence

	Total	Urban	Rural
Total fertility rate			
1992	6.5	5.8	7.1
1996	6.1	5.1	6.9
2001–02	5.9	4.3	6.9
CPR, all methods, %			
1992	15.2	20.8	10.3
1996	25.9	33.3	20.9
2001–02	34.2	45.7	27.9
CPR, modern methods, %			
1992	8.9	15.3	3.2
1996	14.4	23.6	8.2
2001–02	25.3	41.2	16.6

Table 2 describes the basic socio-demographic characteristics of the full sample of women surveyed in the 2001–02 Zambia DHS (column 1) and the characteristics of the study sample (column 2). The study sample includes women who were fecund and sexually active. Nearly half of the women surveyed were younger than 24, and about one-third were between the ages of 25 and 34. However, the study sample included a smaller percentage of women under age 24 than the full sample. The majority of women in both samples finished primary school but did not continue with secondary school. About two-fifths of women in the full and study sample lived in urban areas. Women in the study sample were slightly less likely to be in the highest socioeconomic quintile than women in the full sample (23 percent vs. 25 percent). Copperbelt and Lusaka, the two most urban provinces, contributed the largest percentage of women in both groups. Combined, the percentages from these provinces approximate the percentage of urban residents in the full sample. About three-quarters of the women in both samples were Protestant, and most of the remainder was Catholic. Seventy-four percent of the study sample was currently married or in a union, compared to 61 percent of the full sample. The majority of women had at least one child. Most of the factors that differentiate the study sample from the overall sample are related to selecting a group of women for the study sample who were the most fertile and in need of family planning.

**Table 2:** Socio-demographic characteristics of women in the full sample and the study sample, Zambia 2001/02

	Full sample (n = 7,658)	Study sample* (n = 4,927)
Percentage of sample	100.0	47.2
Age		
15–24	45.4	39.3
25–34	30.7	35.4
35–39	10.0	11.3
40–44	7.9	8.3
45–49	6.1	5.7
Educational attainment		
No education	12.1	13.0
Primary	58.0	60.0
Secondary and higher	30.0	27.0
Urban	40.1	38.2
Currently working	54.6	57.6
Socioeconomic status**		
Very low	18.8	19.1
Low	18.2	18.3
Medium	18.8	20.0
High	18.8	20.0
Very high	25.4	22.6
Religion		
Catholic	23.0	22.4
Protestant	75.3	75.6
Muslim	0.3	0.3
No religion/other	1.5	1.8
Marital status		
Never married	24.8	14.0
Married/cohabiting	61.4	74.0
Formerly married	13.9	12.0
Number of children		
None	27.0	15.2
1	17.4	20.1
2	14.3	16.7
3	12.0	13.8
4+	29.3	34.2

* Includes women who are fecund and sexually active; weighted percentages and unweighted n's presented. Some n's smaller than total due to missing data.

** The distribution of women across quintiles is not exactly 20 percent after taking into account the survey design and weighting of the sample.

Table 3 shows the family planning characteristics of women in the study sample by residence. About one-third (34 percent) of women in the study sample used contraception, either modern or traditional methods; 23 percent used modern methods. Among users, 69 percent used modern methods. The most common method used by women in Zambia is the oral contraceptive pill. Ideal family size was high in Zambia with about half of women wanting at least five children (not shown). When asked if and when women want more children, about one-third of women wanted no more children. One-quarter of women wanted to space births an interval greater than two years. The overwhelming majority of women (85 percent) approved of family planning. Finally, only a small minority (10 percent) of women received a household visit from a community health worker within the prior 12 months.

**Table 3:** Percentage of women in the study sample by selected family planning characteristics and type of residence, Zambia, 2001/02

	Study sample*		
	
	Total (n = 4,927)	Rural (n = 3,365)	Urban (n = 1,562)
Contraceptive use **	34.4	28.7	43.7
Using modern methods**	23.2	14.7	37.0
Type of method among users **			
Modern			
Pill	33.6	24.9	42.4
Injectables	12.6	9.3	16.0
Condom	16.3	14.7	17.9
Sterilization	5.3	3.5	7.2
Other modern	1.4	0.4	2.3
Traditional			
Withdrawal	13.3	21.5	4.9
Periodic abstinence	3.5	4.2	2.9
Lactational amenorrhea	7.1	9.3	4.7
Other traditional	7.0	12.1	1.7
Desire for more children **			
Wants more soon	22.7	25.4	18.4
Wants more later	32.6	34.4	30.0
Wants more, unsure of timing	9.7	8.5	11.5
Undecided/missing	2.4	2.9	1.6
Wants no more	32.6	28.8	38.8
Health worker visit last year **	10.3	11.9	7.6
Heard family planning on radio recently **	46.5	33.4	67.7
Approves of family planning **			
No	10.4	13.1	6.0
Yes	85.2	81.1	91.8
Don't know/missing	4.4	5.8	2.2
Amenorrheic	28.8	36.0	17.1

* Includes women who are fecund, sexually active, and not currently amenorrheic. Weighted percentages and unweighted n's presented. Some n's smaller than total due to missing data.

** Significant difference between urban and rural women at 5 percent level, using Pearson's χ^2 ^test.

Family planning characteristics vary by type of residence, according to Table 3. Women living in urban areas are far more likely to use modern methods than rural women (37 percent and 15 percent, respectively) whereas women using family planning from rural areas are more likely to use traditional methods (47 percent of rural users vs. 14 percent of urban users). About 12 percent of rural women received a household visit from a community health worker in the last year, compared to almost 8 percent of urban women. This difference was significant. A significantly greater proportion of urban women approved of family planning (92 percent) compared to rural women, but even among rural women, four out of five women approved (81 percent).

### Logistic regression

The odds ratios from the logistic regression of factors associated with modern use of family planning (vs. traditional use or no use) are presented in Table 4 for all women and then stratified by place of residence. In models that compare any method use to non-use, the findings (not shown) are similar to those presented in Table 4. Women from urban areas were 1.56 times (95 percent CI: 1.24–1.96) more likely to use modern methods than women from rural areas, controlling for all other factors in the model. Women who are age 35 and older were significantly less likely to use any family planning method than sexually active and fecund women ages 15–24. This finding was similar among the urban stratified sample but in the rural sample, even women 25–34 were significantly less likely than 15–24 year olds to use modern family planning. Among all women and women in both rural and urban areas, the number of living children was significantly associated with modern family planning use such that women with only one child were twice as likely to use family planning as women without children (OR: 2.07, 95% CI: 1.48–2.89). Other consistent results across both urban and rural areas included education level (women with less education were less likely to use family planning); desire for children (women who wanted to delay [OR: 3.34, 95% CI: 2.53–4.41] or limit [OR: 3.52, 95% CI: 2.64–4.71] were more likely to use than women who wanted children soon); and the respondent's approval of family planning (women who approved of family planning were significantly more likely to be users of a modern method [OR: 5.87, 95% CI: 3.37–10.24]). A separate analysis (not shown) of women in union examined women's perceptions of their husband or partner's approval of family planning. Compared to women who did not perceive husband approval of family planning, those who perceived approval were more likely to use modern methods.

**Table 4:** Multivariate logistic regression odds ratios and confidence intervals for analyses of modern method use, total and stratified by type of residence, Zambia 2001/02

	Total (n = 4,927)		Rural (n = 3,365)		Urban (n = 1,562)	
	
	OR	95% CI	OR	95% CI	OR	95% CI
Urban	1.56	1.24–1.96				
Health worker visit last year	1.37	1.00–1.86	1.83	1.29–2.58	0.82	0.45–1.49
Age						
15–24 (r)						
25–34	0.88	0.67–1.15	0.60	0.41–0.87	1.14	.078–1.68
35–39	0.46	0.31–0.66	0.48	0.29–0.79	0.37	0.21–0.65
40–44	0.25	0.16–0.38	0.18	0.10–0.31	0.31	0.15–0.67
45–49	0.08	0.04–0.15	0.08	0.04–0.16	0.07	0.02–0.20
Marital status						
Never married (r)						
Married/cohabiting	1.93	1.34–2.77	0.95	0.61–1.48	3.88	2.25–6.67
Formerly married	0.69	045–1.07	0.43	0.26–0.72	1.01	0.53–1.90
Currently working	0.98	0.83–1.15	0.83	0.66–1.05	1.15	0.91–1.44
Socioeconomic status						
Very low	0.54	0.39–0.74	0.36	0.20–0.62	0.53	0.36–0.78
Low	0.54	0.40–0.74	0.39	0.26–0.61	0.61	0.36–1.06
Medium	0.59	0.44–0.78	0.43	0.27–0.66	0.60	0.42–0.86
High	0.59	0.44–0.78	0.39	0.26–0.59	0.72	0.47–1.10
Very high (r)						
Number of living children						
0 (r)						
1	2.07	1.48–2.89	3.11	1.88–5.15	1.61	1.00–2.61
2	2.53	1.69–3.79	3.96	2.38–6.59	1.87	1.02–3.45
3	3.22	2.08–4.99	5.44	2.93–10.10	2.46	1.31–4.59
4+	3.81	2.42–5.99	6.25	3.30–11.89	3.26	1.65–6.44
Educational attainment						
No education	0.38	0.26–0.56	034	0.21–0.53	0.67	0.28–1.62
Primary	0.65	0.53–0.81	0.69	0.50–0.96	0.29	0.44–0.79
Secondary and higher (r)						
Desire for more children						
Wants more soon (r)						
Wants more later	3.34	2.53–4.41	2.85	1.94–4.17	4.22	2.79–6.38
Unsure/missing	2.89	1.95–4.30	1.54	0.89–2.64	5.67	3.18–10.09
Wants no more	3.52	2.64–4.71	3.02	1.99–4.58	3.81	2.55–5.70
Heard family planning on radio recently	1.20	0.99–1.45	1.64	1.30–2.07	0.84	0.63–1.13
Approves of family planning						
No (r)						
Yes	5.87	3.37–10.24	6.13	2.81–13.41	5.43	2.30–12.81
Undecided/missing	2.55	1.21–5.37	0.99	0.28–3.51	7.77	2.46–24.56
Amenorrheic	0.15	0.12–0.18	0.17	0.13–0.23	0.12	0.08–0.18

All analyses are weighted and control for survey design. Unweighted sample sizes are presented.

Weighted sample sizes are: 4,906 (total); 3,034 (rural); and 1,872 (urban).

The main urban-rural distinction in Table 4 is that only among women in rural areas was a household visit by a community health worker associated with modern use of family planning (p < 0.05). The relationship for urban women was not significant. Rural women who were visited by a health worker were 1.83 times (95% CI: 1.29–2.58) more likely to currently use a modern method than women who were not visited by a health worker, controlling for all other variables. In models that included the full study sample and an interaction for place of residence and health worker visit, the results were the same as those discussed based on the stratified models (not shown). Another urban-rural difference was exposure to family planning messages on the radio, which was significantly associated with modern use in the rural sample (OR: 1.64, 95% CI: 1.30–2.07) but not in the urban sample (OR: 0.84, 95% CI: 0.63–1.13).

### Simulations

A simulation determined the effect of all women receiving at least one household visit from a health worker if all other variables were held constant. This is of particular interest in rural areas where household visits by health workers may be the main access point for women to learn about family planning use and receive family planning services. Compared to 14.7 percent of women from rural areas who currently use modern methods, family planning use rates would rise to 20.6 percent (a 5.9 percentage point increase) if all rural women's households had been visited by a health worker, holding all other factors constant. The rate is predicted to decrease in urban areas by about 3 percentage points if all urban women received a visit owing to the odds ratio being less than one (insignificant) for household visits and modern use among urban women (Table 4).

Household visits by community health workers are intended to improve access to family planning services. Similar simulations to the one described above were performed for family planning characteristics oriented more toward demand creation, in order to compare each factor's predicted change in modern contraceptive use compared to health worker visits. Three "demand-side" factors were tested: a desire to delay future childbirths, a desire to limit future births, and exposure to family planning messages on the radio. If all rural women changed their fertility desire to wanting to delay future births, in rural areas the CPR for modern methods would increase by 8.0 percentage points. If all rural women changed their fertility desire to wanting to limit additional births, the modern CPR would increase by 9.6 percentage points. Compared to the predicted effect for family planning household visits, the predicted effects associated with fertility desires are of a larger magnitude. If all rural women heard at least one family planning message on the radio in the past year, the modern CPR in rural areas would increase by 3.1 percentage points, about half the effect associated with a family planning household visit for every woman.

## Discussion

The contraceptive prevalence rate in Zambia for urban women was significantly higher than the contraceptive prevalence rate for rural women, even after controlling for selected individual and family planning characteristics associated with living in an urban area. Family planning outreach programs have aimed to close this gap by providing information and contraceptive supplies to women and men, particularly in rural areas. These outreach efforts in Zambia were associated with higher modern contraceptive use among rural women. If all households in rural Zambia received a home visit from a community worker, the contraceptive prevalence rate for this group would increase by 5.9 percentage points; this amounts to a 21-percent increase in modern contraceptive use and represents a substantial improvement. Nevertheless, the gap between rural and urban patterns in contraceptive use (and TFR) would remain. Moreover, changes to fertility desires of rural women would increase use of modern family planning more than would a household visit by a health worker to each rural woman.

Our findings are fairly consistent with the existing literature. Previous studies have noted similar differentials in contraceptive use between urban and rural residents in sub-Saharan Africa [1,6]. In addition, while several quasi-experimental studies have found positive associations between contraceptive use and community-based distribution programs [7,9,26,27], the size of these associations varies widely due in part to differences in study design and setting.

This study has several limitations. First, it relies on cross-sectional data that limit our ability to determine whether and how the community health worker visit affects family planning use. Longitudinal tracking of Zambia's CBD efforts would provide a more complete picture of how individuals change their behavior in response to family planning outreach. Second, a simple measure of whether a community health worker visited the household may not be a robust predictor of overall family planning outreach within Zambia. For example, the quality and the frequency of the field worker visits may matter [28], but these were not measured. Furthermore, the measure of outreach used in this study is not specific to family planning. Community health workers can visit a household for numerous reasons unrelated to family planning use. Had the measure of outreach been specific to family planning, the estimated rise in contraceptive use if all women received a household visit may have been higher. Third, other forms of outreach, such as employer programs and social marketing programs, may confound our attempt to isolate the effect of household visits. Finally, there are a number of unobserved factors that influence access (measured through place of residence and health worker visit) that are also associated with modern contraceptive use, introducing a problem of endogeniety, which could bias the association between a health worker visit and contraceptive use. A common strategy to control for endogeniety is instrumental variable methods that require identifying variables (instruments) associated with the health worker visit but not associated with contraceptive use. Unfortunately, in the DHS, no appropriate instrumental variables were available. Therefore, the analyses are limited by the available data. Future studies should collect information on potential instruments such as whether the health worker lives in the village and whether the informant knows the health worker; these measures would be useful to reduce the potential for endogenity problems.

## Conclusion

Family planning programs allocate resources based on implicit decisions about the relative importance of supply-side and demand-side approaches for affecting contraceptive use and fertility. In other words, program planners decide if interventions should focus on improving access to contraceptives and/or on changing the fertility desires and motivations of potential users. A look at data from Zambia offered an opportunity to learn more about the impact of community-based distribution as a supply-side strategy on increasing uptake of family planning services. Proponents of CBD argue that home visits made by community workers may help to fill an unmet need for family planning among women who lack ready access to contraceptive supplies and information. Sceptics of CBD point to research demonstrating that cultural and social barriers to using contraception may determine whether a woman with an unmet need actually uses family planning to a greater degree than does access [29].

Our model indicates that community-based outreach, as measured here and as it existed in Zambia in 2001–02, would lead to only a modest increase in contraceptive use. Supply provision appears to be one of many determinants of family planning use in Zambia. Creating demand may be at least as important [30,31]. This approach includes targeting women and men to increase approval of family planning. An unmet need for family planning may exist in rural areas; however, providing contraceptives may not reduce this unmet need if women and their partners a) do not approve of family planning, b) lack firm desires to delay or limit childbearing, or c) do not find that the available methods meet their needs.

The World Health Organization, with support from bilateral organizations including the United States Agency for International Development (USAID), established a framework in 2004 to "reposition" family planning in sub-Saharan Africa. This initiative recognizes the attention deficit paid to family planning as a result of competing priorities, most notably the HIV/AIDS epidemic [5,32]. The initiative called for better access to family planning services at all levels, including at community-based outlets. The results from our analysis suggest that the repositioning effort should promote community-based distribution with a component that aims to generate demand for family planning in rural areas through changing community norms for large families and increasing male and female approval of family planning. Strategies need to address issues of access while attempting to communicate how family planning services can help users realize their fertility intentions. Casterline and Sinding (2001) highlight three factors that, along with lack of service provision, contribute to unmet need [31]. Based on an empirical review of the evidence, Casterline and Sinding conclude that there is: social opposition to family planning use, inadequate knowledge about contraceptive methods, and health concerns about possible side effects [31]. Family planning programs should design interventions that address these issues, with a focus on satisfying unmet need and on changing the cultural and individual beliefs and fertility desires of potential users. Specific ways in which governments and program managers can induce demand for contraception include: exposure to family planning messages through mass media, community mobilization events that seek to increase male support for family planning, and engagement of community opinion makers. The Navrongo project in northern Ghana sets a laudable precedent [13,33]. It paired the community placement of trained nurses with the involvement of traditional leaders and male volunteers, a combination that, from 1993 to 1999, led to a 15-percent decline in fertility compared to comparison communities [13]. Until donors and program architects (re)invest sufficient capital in family planning demand creation and supply of services, progress in sub-Saharan Africa will continue to lag behind improvements experienced in other geographic regions. Fortunately some policymakers have renewed their commitment to family planning programs in sub-Saharan Africa as part of the repositioning initiative.

## Competing interests

The author(s) declare that they have no competing interests.

## Authors' contributions

ISS conceived of the study, designed the study, reviewed data output, and edited drafts of the manuscript. JSW conducted the data analysis, wrote the initial draft of the manuscript, and revised subsequent drafts. All authors read and approved the final manuscript.

## Pre-publication history

The pre-publication history for this paper can be accessed here:


